# Languages in Drier Climates Use Fewer Vowels

**DOI:** 10.3389/fpsyg.2017.01285

**Published:** 2017-07-27

**Authors:** Caleb Everett

**Affiliations:** Department of Anthropology, University of Miami, Coral Gables FL, United States

**Keywords:** phonetics, environment, adaptation, psychological, language, evolution

## Abstract

This study offers evidence for an environmental effect on languages while relying on continuous linguistic and continuous ecological variables. Evidence is presented for a positive association between the typical ambient humidity of a language’s native locale and that language’s degree of reliance on vowels. The vowel-usage rates of over 4000 language varieties were obtained, and several methods were employed to test whether these usage rates are associated with ambient humidity. The results of these methods are generally consistent with the notion that reduced ambient humidity eventually yields a reduced reliance of languages on vowels, when compared to consonants. The analysis controls simultaneously for linguistic phylogeny and contact between languages. The results dovetail with previous work, based on binned data, suggesting that consonantal phonemes are more common in some ecologies. In addition to being based on continuous data and a larger data sample, however, these findings are tied to experimental research suggesting that dry air affects the behavior of the larynx by yielding increased phonatory effort. The results of this study are also consistent with previous work suggesting an interaction of aridity and tonality. The data presented here suggest that languages may evolve, like the communication systems of other species, in ways that are influenced subtly by ecological factors. It is stressed that more work is required, however, to explore this association and to establish a causal relationship between ambient air characteristics and the development of languages.

## Introduction

The communication systems of many species are known to be ecologically adaptive, being impacted by factors such as humidity (Wilkins et al., 2013). Such adaptivity is not traditionally thought to characterize human speech, however. This position of linguistic “autonomy” has now been called into question, however, by studies pointing to potential environmental influences on speech sounds. (Munroe et al., 2009; Everett, 2013; Everett et al., 2015) Such studies have confronted strong objections, in part (and to varying degrees) because of their utilization of binning strategies through which linguistic and/or geographic variables were categorized. The present study avoids such binning and offers, via several analytical methods, evidence of an association between two continuous variables: ambient humidity and vowel utilization. I suggest that this association may be motivated by the influence of dry air on the vocal folds, though more research is required to show that this influence motivates the distribution described here and in other work on this topic (Everett et al., 2015). The association between ambient humidity and vowel utilization is, we will see, unlikely due to potentially confounding factors like linguistic phylogeny or contact between languages. While I avoid strong claims of causality, I conclude that the uncovered association merits further inquiry. In other words, the association does not demonstrate that languages adapt to ecological factors, it simply suggests that idea deserves continued consideration.

Languages with complex tonality are apparently less likely to develop in desiccated regions. In previous work, colleagues and I have argued that this distributional pattern is possibly due to subtle diachronic pressures resulting from the heightened difficulty of maintaining precise pitch when vocal folds are consistently exposed to desiccated air (Leydon et al., 2009; Everett et al., 2015). Laryngology studies do suggest that phonation/voicing is affected by dry air. In one study it was observed that the effects of dry air include increased jitter rates that may impact the production of precise pitch (Hemler et al., 1997). Yet it is still debated whether such minor effects on jitter rates actually impact pitch production in normal speech, and whether languages with complex tone really do rely on more precise pitch patterns in the speech stream (De Boer, 2016; Everett et al., 2016a,b). What is less debatable is that research in laryngology has shown the desiccation of vocal cords leads to greater perceived phonatory effort on the part of speakers and that laryngeal desiccation impacts the viscoelasticity of the vocal folds. [See the survey of some relevant findings by Leydon et al. (2009).] For instance, in a recent experiment with elderly speakers, it was observed that increasing ambient humidity to moderate levels reduced the perceived phonatory effort and vocal tiredness reported by those speakers in a loud-reading task (Sundarrajan et al., 2017). The effects uncovered in such studies surface despite relatively limited exposure to desiccated ambient air, in contrast to populations living in very arid environs. The salutary effects of humidity on phonation could help explain the pervasive pattern reported here. Future work could explore this possible connection with other methods, including experimental ones.

In addition to the association between less tonality and aridity, other correlations between geography and phonemic inventories have been observed. These include the greater frequency of ejective sounds in high elevation regions (Everett, 2013) and the higher rate of consonant-vowel syllables in languages in warm regions (Ember and Ember, 2000, 2007; Fought et al., 2004; Munroe and Silander, 2009). It is suggested below that all these correlations are interrelated and, if causally motivated by the environment, may have one underlying motivator. Despite such associations, many language researchers remain skeptical of any meaningful relationships between ecological factors and human phonologies. Since language is transmitted socially, it is still unclear how ecological factors may come to influence phonetic patterns. In previous work colleagues and I have suggested tentative mechanisms through which some effects may surface, but the likelihood of these mechanisms is admittedly open to debate (Everett et al., 2016a,b). Despite such debate, some scholars now seem open to the possibility that languages are impacted by ecology. The more general suggestion that language-external factors impact language is evidenced in other contemporary research as well, for instance in work showing a negative correlation between population size and morphological complexity (Lupyan and Dale, 2010, 2016).

In short, the last decade has seen the publication of a variety of studies hinting that, contra traditional linguistic dogma, languages develop in ways that are sensitive to ecological pressures. Yet the relevant studies on linguistic sounds share a characteristic that some scholars have found problematic: the simple binning of languages by linguistic or ecological characteristics. [This is not true of all work examining external influences on language, however, see Lupyan and Dale (2010).] For instance, one recent study suggested that languages rely on consonants more in cold regions with less vegetation (Maddieson and Coupé, 2015). The observed correlation was found after languages were binned into categories such as “consonant-heavy,” meaning that a language’s ratio of consonant phonemes to vowel phonemes is high. Yet there is arguably no clear independent motivation for marking the divisions between the created categories in this and other studies. For instance, in Everett et al. (2015), colleagues and I categorized languages as having or not having “complex tonality” in order to facilitate the testing of a specific hypothesis. Yet, as we were aware, languages vary dramatically and non-discretely in the extent to which they rely on pitch for contrasting meaning, and on the extent to which precise pitch is used for other purposes. (Ladd, 2016) Such binning strategies are generally the result of methodological exigencies and the limitations of extant databases but, at least to some scholars, they minimize the inferences that can be drawn from the associations in all work so far undertaken on this topic. A similar observation may be made with respect to ecological factors, which have also been binned to facilitate the grouping of environments and test for geo-phonetic patterns. In some research, populations of speakers have been grouped as living in either “cold” or “warm/moderate” climates (Munroe et al., 1996). In a study of mine, languages were categorized dichotomously as being native to either high or low altitude regions, for a portion of the analysis (Everett, 2013). For another portion of the analysis, the altitudes of language locales were analyzed continuously, but some of the objections to the study have centered around the strategy employed for the discrete binning of language locales according to elevation regions. There is a concern that the observed correlations in such studies might have benefited from the placement of the category divisions (Dediu et al., 2017). One could debate whether this concern has been exaggerated, but it must be acknowledged that, to date, all studies on this particular topic have relied to varying degrees on the discontinuous grouping of language locales and/or language types into two or a few categories. The results of such studies face resistance, at least in part, because of this methodological tack. So a central aim of the present study is to test for a key ecological-linguistic correlation while relying entirely on continuous data.

Despite this shared methodological tack, the recent studies on this topic certainly intimate that human language may be ecologically adaptive. Alternate explanations for most of the associations are still missing, beyond pointing to the well-known existence of spurious correlations. Yet it is also well-known that the uncovering of correlations is a key tool in the scientific arsenal, as they frequently point to relationships that merit further inquiry. Since languages exhibit a bias toward ease of articulation and some sound patterns may be more easily (even slightly) produced under certain ambient conditions, some language researchers now seem open to considering the possibility of ecological influences on speech sounds. It would appear that a clearer understanding of this issue is a desideratum for the language sciences (Evans, 2016; Greenhill, 2016). In an effort to contribute to that understanding, this study explores the association of human sound systems and their ecologies. It is the first to do so without relying on binning strategies. Languages are not grouped according to any phonetic/phonological categories, and ecologies are not discretely categorized either. Additionally, the study relies on the largest data set so far considered in such work. Analysis of that data set demonstrates that vowels are relatively less frequent (when contrasted to consonants) in languages in dry regions. This pattern appears consistent with laryngology evidence suggesting that phonation/voicing threshold pressure and perceived phonatory effort are heightened by inhaled dry air and the superficial dehydration of vocal cords (Sivasankar and Fisher, 2002; Leydon et al., 2009). It is also consistent with the recent finding that perceived phonatory effort and perceived tiredness are mitigated, amongst elderly speakers, when ambient humidity is increased (Sundarrajan et al., 2017). Since languages are biased toward less articulatory effort (Napoli, 2014), it is at least possible that they could be impacted by the heightened laryngeal challenges associated with the inhalation (especially oral inhalation) of dry ambient air. This possibility is difficult to evaluate conclusively given the many complex factors at work in language change, but I argue that it nevertheless merits further investigation.

The linguistic variable investigated here, the rate at which languages rely on vowels compared to consonants, was selected for four reasons. First, it has been suggested for some time now that languages in colder regions rely less on vowels. This suggestion was initially based on binned data with small samples, or without controlling for Galton’s problem (Munroe et al., 1996; Maddieson et al., 2011). So the findings presented here relate to previous results, but address the issue with a novel approach and larger data set. Second, the linguistic variable investigated here relates to all spoken human languages. All rely on vowels, sonorant voiced sounds produced with oral aperture. Some of the previous work on this topic has focused on linguistic phenomena like complex tone and ejectives that, while not rare, only occur in a subset of the world’s languages. Third, the variable considered here has a clearer potential connection to experimental work in laryngology. One issue with investigations of ecological adaptation in speech is that myriad *post hoc* explanations for uncovered correlations may be possible. [See the debate in Ember and Ember (2000) and Munroe et al. (2000).] So the linguistic variable selected should have some connection to prior nonlinguistic research. In the aforementioned study on tonality, we connected tonality to findings in laryngology suggesting that complex pitch production may be more difficult to achieve in arid regions. Yet, while little of the laryngology research relates to pitch (De Boer, 2016), a more common finding is that dry air increases perceived phonatory effort since vocal cord usage becomes slightly more effortful after the inhalation of dry air (Erickson and Sivasankar, 2010). Such effects surface even after short exposures of the larynx to dry air. Of course the healthy human larynx is capable of achieving homeostasis and adapting to environmental pressures, so these effects may not be felt in all individuals equally and may only surface in minor ways during real-world speech situations. Yet even minor effects could potentially yield, over the long haul, functional pressures on speech. So a simple possibility exists: Languages in dry regions may exhibit a bias toward less vocal cord usage. Vowels require voicing and are the sounds that generally carry stress, which often requires greater amplitude of vocal cord vibration. Given such factors, it is worth considering whether there is a slight bias against the utilization of vowels in dry places. Since vowels are critical to the audibility of language, any vowel-reductive patterns would likely be minor. While many consonants are also voiced, many are not and consonants’ degree of voicing (i.e., voice-onset-time) can vary substantially. The database relied on here does not encode voicing status for all consonants, creating further motivation for focusing on vowels. Nevertheless, some analysis of voicing in consonants is presented after the main analysis of vowels.

Fourth and finally, the linguistic variable used in this study was selected because it yields continuous data derived from transcriptions of actual words, as opposed to being derived from lists of phonemes. Phonemic inventories, which have been used in all previous studies on this topic, are actually not ideal bases for investigating potential ecological interactions of the sort being considered here. After all, they are only indirect representations of which sound patterns are most characteristic of a language, since any sound that is used in a semantically contrastive function, i.e., in a minimal pair, is included in a language’s phonemic inventory regardless of the phone’s frequency. So, for instance, if we were ascertaining the consonant-to-vowel (C:V) ratio of the English phonemic inventory, all consonants and vowels would carry the same weight even though it is well known that some sounds are much more common than others in speech. High C:V ratios demonstrate relative diversity of consonant types, rather than actual heightened reliance on consonants in speech. To get a sense of which sounds and sound patterns are actually most characteristic of English or another language, we have to have some way of determining the relative frequency of sounds. The specific linguistic variable introduced below, “vowel index,” allows for such a determination. (In the “Discussion” section, I examine the relationship between vowel index and C:V ratio.)

The selection of the main ecological variable relied on here, specific humidity, is also well motivated. Specific humidity refers to the ratio of water in the air (See “Materials and Methods”). Epidemiological, laryngological, and anthropological studies have demonstrated that dry ambient air, particularly very dry air, has pervasive effects on the human body. These effects include increased prevalence of xerostomia, laryngitis and other vocal-tract maladies, the effects of reduced humidity on the evolution of cranial morphology, and the aforementioned effects on phonation (Sivasankar and Erickson-Levendoski, 2012; Maddux et al., 2016). The latter effects are exacerbated by oral breathing that is promoted by nasal blockage that is more prevalent in dry and particularly cold-dry conditions (Mäkinen et al., 2009).

**FIGURE 1:**
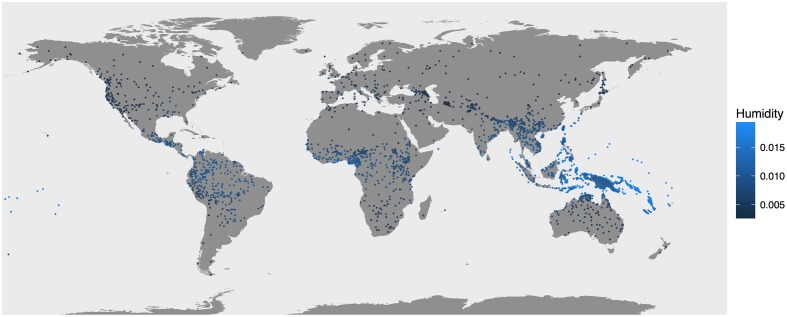
Locations of the language varieties represented in the main analysis.

Using the continuous phonetic and ecological variables selected, the potential interaction of languages and ambient air was tested. This study relied on data from the Automated Similarity Judgment Program (Wichmann et al., 2016). The creators of the database employ a transcription system that conveys characters of the International Phonetic Alphabet with typewritten letters, with a sufficient degree of specificity as to count instances of consonants and vowels. At present the database contains phonetic transcriptions of 7221 word lists based on the analysis of many written sources including work by linguistic fieldworkers. Word lists for constructed languages and proto-languages were excluded from analysis. Some languages are represented by more than one list as multiple dialects are represented. Given the cline-like nature of the language/dialect distinction, I refer to the word lists as representing separate “language varieties” and rely extensively on methods that control for the over-representation of any language families. Each language variety is categorized according to its linguistic family as labeled in the WALS database (Dryer and Haspelmath, 2016). For each of the word lists the incidence of vowels, compared to the total transcribed vowels and consonants, was calculated. The calculation of vowels as a ratio of all sounds is referred to as the “vowel index” of each language variety. (Terms like “vowel ratio” or “consonant-to-vowel ratio” are avoided since they serve other functions in linguistics.) Word lists in the ASJP database have coordinates representing the locales to which their represented languages are thought to be native. These locales, even if not exact representations of ancestral homelands, approximate the appropriate regions. For the relatively few widespread languages, the coordinates denote the locale thought to be associated with the language’s development, e.g., southeast England for English. For 4012 language varieties in the database, specific humidity rates were obtained for their presumed locales, by cross-referencing the varieties with the humidity data in Everett et al. (2015). Of course, populations of speakers do not reside in the exact same location long-term, and climatic patterns also change over the long-term (Moran, 2016). Still, the mobility of most populations is relatively confined geographically (and cultures are typically well-adapted to particular environs) and certain climatic patterns hold regardless of weather cycles. Equatorial regions tend to be hotter and more humid, high elevations and deserts are arid, and so forth, regardless of climatological undulations. Furthermore, most major geographic factors, e.g., the Sahara and Amazonia, existed long before the languages on which this analysis is based. In short, relying on such climatological data seems the best approach available to explore this issue. Mean annual temperature data were ascertained for 6901 language varieties. Temperature is an (imperfect) proxy for specific humidity because air at colder temperatures can “hold” less water (Maddux et al., 2016). Basic tests were conducted on the larger word-list set with temperature data. Their results, some presented in the “Materials and Methods” section, are consistent with the findings discussed next, though generally less robust. This suggests that any associations with temperature may be epiphenomenal. The 4012 language varieties used in the main analysis, along with their locales’ associated humidity values, are presented on the map in **Figure 1**.

The transcriptions utilized represent 40 words denoting basic semantic concepts. These include body parts, pronouns, common animals, frequent actions, as well as natural entities like ‘water’ and ‘sun.’ Some lists in the database have more than 40 words or a few less. The word lists are excellent data for testing any potential interactions with non-linguistic variables, since the words are not readily susceptible (though not immune) to contact-based effects. Some of these words are amongst the most common words in language, making them good indicators of how languages rely on particular sounds (Calude and Pagel, 2011). Recall that phonemic inventories, on which most studies of this topic have relied, do not actually offer information about the relative frequency of sounds in a given language.

## Results

**FIGURE 2:**
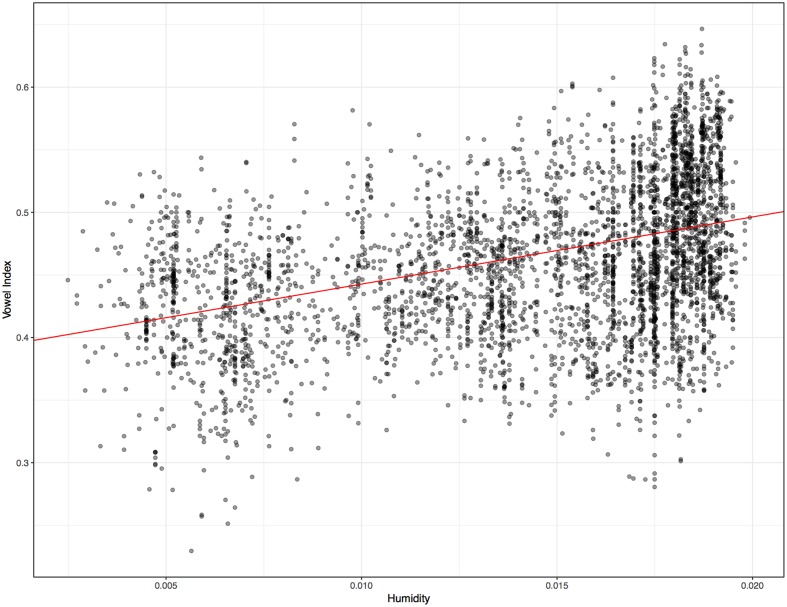
Vowel index rates for the 4012 word lists, with associated specific humidity values. The line has a slope of 5.37 with a *y*-intercept of 0.389. For the simple regression: F-statistic = 756.2 on 1 and 4010 DF, *p* = 0.000. *R^2^*: 0.159. A Box Cox transformation was applied to the data, to ensure homoscedasticity. The estimated lambda values was 0.9. With the fudge factor no transformations were required.

For the 4012 word lists used in the core analysis, vowel indices ranged from 0.230 to 0.647, with a median of 0.458. The median for the larger set of 6901 lists was also 0.458. Languages aggregate around a rate of one vowel for every consonant. While it is known that CV syllables are quite common in languages (Maddieson, 2013), these figures represent, to my knowledge, the first quantification of the relative frequency of vowels and consonants across a major cross section of the world’s languages. Some previous work has examined the relative frequency of vocalic and consonantal *phonemes* in much smaller sets of languages. For instance, Yegerlehner and Voegelin (1957) found, via text analysis, that the median ratio of vocalic phonemes across nine languages was 0.475. Such phoneme-based ratios evident in texts are similar to the vowel indices obtained here, though vowel indices are phonetically rather than phonologically based. Consider the two most extreme cases in Yegerlehner and Voegelin: The highest vowel ratio in their set was obtained for Maori, at 0.587. The vowel index obtained here for Maori is 0.559. The lowest vowel ratio obtained in Yegerlehner and Voegelin’s small sample was for Navajo, at 0.44. The vowel index obtained for Navajo with the present methods is 0.454. So this computationally based analysis of frequent words apparently yields similar results to visual inspections of phoneme counts in texts. In the data considered here, the four language varieties with the lowest vowel indices, ranging from 0.230 to 0.258, are all Salishan languages of the Pacific Northwest. These languages are known for their complex strings of consonants (Flemming et al., 2008). The language with the highest vowel index, 0.647, is the Amazonian isolate Pirahã that is also known to exhibit some unusual phonetic characteristics (Everett, 2005). With respect to the humidity data, the median ratio for specific humidity is 0.0162, with a minimum value of 0.0025 and a maximum of eight times that, at 0.020. This marked disparity is a reminder that, while all people live at the bottom of the same ocean of air, many reside in different seas. Everett et al. (2016a) with respect to temperature, the median annual temperature is 24.2 Celsius. In **Figure 2** the 4012 languages are plotted according to humidity and vowel index.

**FIGURE 3:**
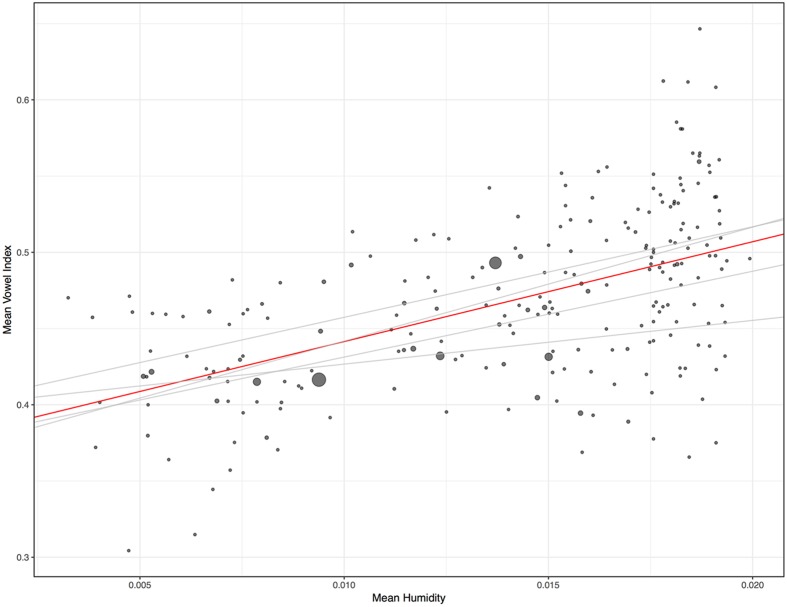
Mean vowel index and mean humidity rates for 229 language families. Lines represent slopes of basic linear regressions. The global trend is represented in red. The red line has a slope of 6.55 with a *y*-intercept of 0.376. For the simple linear regression: F-statistic: 90.77 on 1 and 227 DF, *p* = 0.000. *R^2^*: 0.286. The gray lines represent trends on each of the four main landmasses (Eurasia, Africa, South America, and North America). Dot size represents mean population (log-transformed).

A simple linear regression for vowel index and humidity reveals an interaction (*R^2^* = 0.159, *p* = 0.000), as evidenced by the positive slope in **Figure 2**. Since vowel indices are proportion data technically bounded at 0 and 1, simple linear regression is not the best approach. A more suitable test is beta regression, or regression of logit-transformed vowel indices. This discussion focuses on results for beta regressions, though remarkably similar results obtain for all three sorts of tests. (The proportion data in this case, while technically bounded at 0 and 1, actually occupy a fairly narrow portion of that range). In the case of the global distribution evident in **Figure 2**, a beta regression also reveals a significant interaction between humidity and vowel index (pseudo *R^2^* = 0.158, *p* = 0.000). Nevertheless, the trend in **Figure 2** could be the result of confounds like the preponderance of particular language families in dry regions. One useful approach to control for such confounds is to treat the language families (including isolates) as separate data points, so that each family carries the same weight. The median and mean vowel index and humidity values of each family were ascertained. The means are plotted in **Figure 3**. Controlling for family in this way, the relationship between variables is actually noticeably strengthened. The beta regression for families’ mean vowel indices and humidity values reveals a more striking association (pseudo *R^2^* = 0.286, *p* = 0.000). If we examine the relationship between median vowel index and median humidity values, so that the data points represent actual languages and locales, the heightened association remains (pseudo *R^2^* = 0.267, *p* = 0.000). One might object that such means and medians are misleading for large linguistic families spoken over diverse geographic regions. The most widespread families do not appreciably impact the results in **Figure 3**, however. The 229 families have an average size of 17.5 language varieties, but six families account for almost half of the total of 4012. Austronesian has 869 representative lists, Indo-European has 208, Afro-Asiatic has 185, Niger-Congo has 409, Trans-New-Guinea has 211, and Sino-Tibetan has 186. Also, 181 Australian varieties are grouped together in the database. Removing all these languages leaves us with 1763 word lists distributed across 222 families–7.9 per family, generally representing a restricted geographic region. When the mean vowel index and humidity values of only these 222 families are considered, the regression reveals the same interaction (pseudo *R^2^* = 0.280, *p* = 0.000). When the median vowel index and humidity values of these 222 families are examined, the same interaction is again observed (pseudo *R^2^* = 0.263, *p* = 0.000).

A multiple regression with the mean vowel index (logit-transformed) of each family as a dependent variable, and mean humidity, region (Eurasia, North America, South America, Africa, Australia, and the Pacific), and mean population (log-transformed) as independent variables, still reveals the interaction of humidity and vowel index (*p* = 0.000). Unsurprisingly, perhaps, no effect of population was observed. The interaction of regions and vowel indices is discussed further below.

As is evident in the regression lines in **Figure 3**, the interaction of vowel index and humidity surfaces within the four major landmasses with the greatest variances in climate. (The association does not surface within Australia, a point returned to below.) These landmasses were analyzed separately because of that variance, and because they are geographically rather than linguistically motivated. [See, e.g., Dryer (1989) for one take on the need for such geographic sampling.] For the 23 families of Africa (757 languages), mean familial vowel index and mean familial humidity are strongly associated (pseudo *R^2^* = 0.343, *p* = 0.0005). For the 31 families of Eurasia (934 languages), they are also strongly associated (pseudo *R^2^* = 0.300, *p* = 0.0003). For the 46 families of North America (299 languages), they are associated but not to the same degree (pseudo *R^2^* = 0.117, *p* = 0.01). For the 69 families of South America (407 languages), they are again strongly associated (pseudo *R^2^* = 0.201, *p* = 0.00003). If we consider instead the median vowel index and humidity values, the cross-family association remains evident within each region. It is again significant across all four regions, and once again is weakest in North America (See “Materials and Methods”).

**FIGURE 4:**
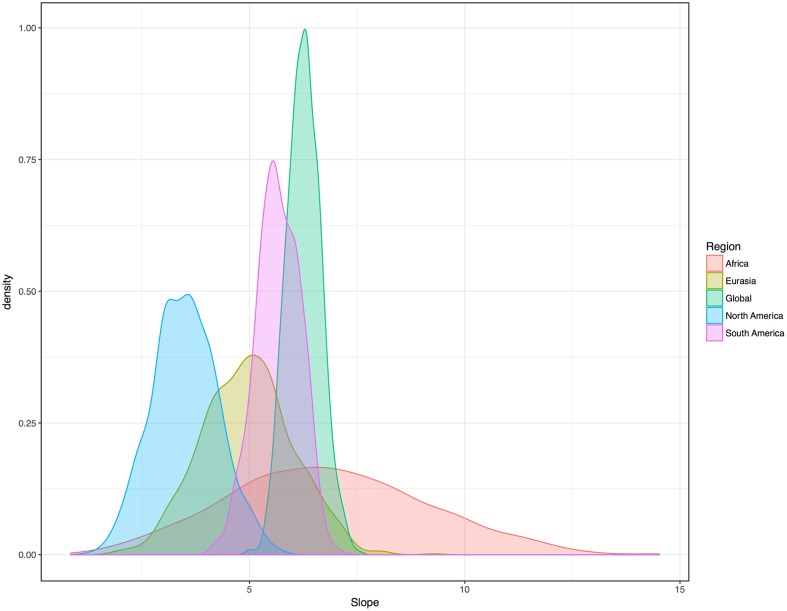
Density distribution (Gaussian kernel) for the slopes of the randomly sampled regressions of vowel indices and humidity. Each color represents 1000 slopes. Each regression is based on a new list created from a sample of languages consisting of one randomly selected member of each family. All 5000 slopes are positive.

To further test the association without relying on medians or averaging, I adapted the method of random sampling used in previous work (Everett et al., 2015). This method also lends equal weight to each family. However, for this study I used random sampling at global and regional scales, so as to control for phylogeny and areal effects simultaneously. For each sample, one language per family was randomly selected and its vowel index and humidity were noted. Then the sample was analyzed with a regression contrasting vowel indices and humidity values. Both linear and beta regressions were used. (Basic linear regression was used simply to test for a positive or negative slope, for each regression.) One thousand regressions of each type were analyzed at the global level, each representing all 229 families. Critically, 1000 tests of each type were also analyzed for each of the four major landmasses with many families, thereby weighting families equally while simultaneously testing regions. The density distribution of slopes for the 5000 linear regressions, offered in **Figure 4**, shows that the pattern is evident within all major landmasses. Once again it shows itself to be weakest in North America. Still, slopes were positive for all 5000 iterations of the simple regressions.

For the beta regressions, 1000 global tests revealed a clear interaction between humidity and vowel index. The mean pseudo *R^2^* value across all 1000 global iterations was 0.23. For Africa, the mean pseudo *R^2^* across 1000 iterations was 0.23. For Eurasia, the mean pseudo *R^2^* across the 1000 tests was 0.19. For South America, the average pseudo *R^2^* was 0.17. For North America, the average pseudo *R^2^* was 0.09. The association was once again found to be positive in all 5000 iterations of the test.

**FIGURE 5:**
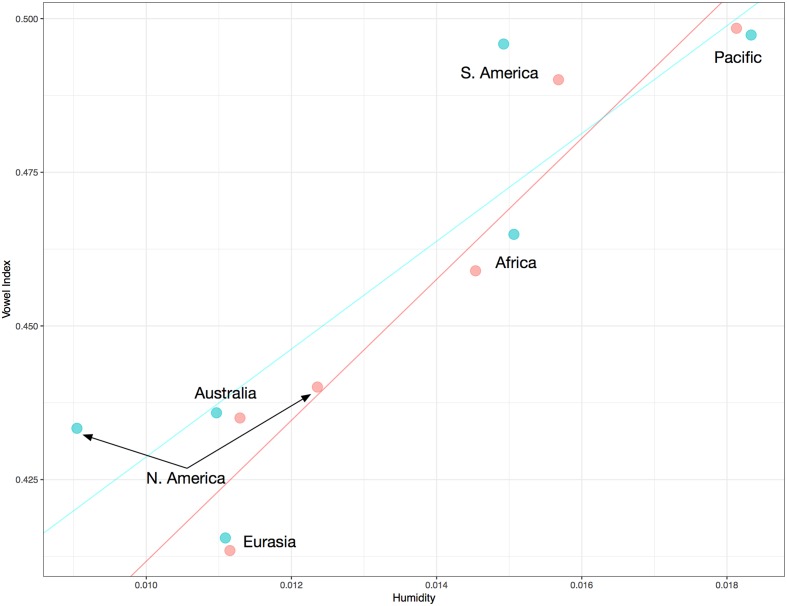
Vowel indices by region, controlled for relatedness. Cyan dots represent the median values of a region, i.e., the median value of the families’ median values. Red dots represent the mean values of the families’ average values, for each region. Beta regressions of either medians or means are significant. (See text.)

The association is evident not just within regions but is also evident across them. We can arrive at the mean vowel index and humidity value for each of the six major “regions” (the four major landmasses plus Australia and the Pacific) by averaging the means of each family in each region. If we then run a beta regression on the regions’ average vowel indices and humidity values, controlling for family in this manner, we find a very striking association between vowel index and humidity (pseudo *R^2^* = 0.917, *p* = 0.000). (If we exclude the Pacific region, since it is not actually a distinguishable landmass, the association changes little: pseudo *R^2^* = 0.902, *p* = 0.000.) If we use the same approach with medians to control for relatedness, we find a similarly striking association between the regions’ vowel indices and humidity values (pseudo *R^2^* = 0.771, *p* < 0.00001). (Again, if we exclude the Pacific region the association remains significant: pseudo *R*^2^ = 0.673, *p* = 0.001.) At least in the case of these six separable areas, regions with lower humidity values tend to have lower vowel indices. This pattern is difficult to reconcile with the idea that the vowel-usage/humidity association is due somehow to contact between languages. While there are a limited number of data points in such a cross-continental correlation, the pattern in **Figure 5** is remarkably consistent with the notion of gradual linguistic adaptation to ecological constraints.

The Pacific “region” is not, of course, a distinct landmass amenable to intra-continental analysis. It consists of many smaller landmasses including New Guinea, Borneo, and Sumatra, as well as many islands that were only relatively recently inhabited through the Polynesian expansion. Yet the “region” is generally characterized by high humidity values with limited variation (See Supplementary Figure 1). This relative environmental consistency motivates its inclusion in the cross-regional rankings just discussed, but is another factor that makes intra-Pacific analyses uninformative. The Australian landmass is a less straightforward case, however. In the database utilized, all language varieties in Australia are grouped together in the same family. Yet, even if we were to categorize them according to the traditional division of Pama-Nyungan/Non-Pama-Nyungan, we would be unable to uncover trends that could be said to characterize the region but not particular families. In contrast, recall that there are between 23 and 69 families represented for each of the four major continents. The Australian data are also problematic in that the Australian landmass does not exhibit the same ecological diversity as the four major continents. The driest regions in the world are extremely cold regions, which Australia lacks. Still, there are some very dry desert regions in Australia and many languages are spoken on the continent. While these regions are not characterized by the extreme aridity observed in the winters of parts of Eurasia, North America, South America, or even Africa, they are still quite dry judging from their annual averages. So a beta regression was run separately for Australia and the results of this regression are inconsistent with the other landmasses. In fact, in Australia a negative association between vowel index and humidity was observed (pseudo *R^2^* = 0.124, *p* = 0.0001). This certainly runs against the general trend and the guiding hypothesis. Still, some caution is required before giving the Australian case equal weight alongside the other continents. In addition to the already-noted issues, another relevant point should be made: The negative correlation in Australia is driven largely by the relatively low vowel indices obtained in higher humidity regions, not by objectively high vowel indices in dry regions. The lowest humidity value obtained for Australia was 0.0050, for three languages. The mean vowel index of these languages was 0.464, very close to the world median of 0.458. In other words, the languages in the dry regions of Australia do not have high vowel indices since they hover around the median for the global sample, while some of the languages in Australia’s more humid regions have lower-than-normal vowel indices. This is worth noting since the hypothesis motivating this work, as in Everett et al. (2015), is that very dry air may impact phonation in at least some real-world contexts. (In that study it was noted that tonality is not observed in Australia.) If this hypothesis is accurate, high vowel indices should be avoided in dry contexts. This expectation is not, strictly speaking, violated in Australia. Consider this: In Australia, the highest humidity value of 0.01775 was obtained for four languages. These languages had an average vowel index of 0.401, which is actually quite a bit lower than the world median. (In contrast, the vowel indices for the languages with the highest humidity values on each of the four major continents were 0.559 [Africa], 0.516 [North America], 0.508 [Eurasia], and 0.496 [South America].) When considered in the light of the values observed in the rest of the world, the Australian trend owes itself to low vowel indices in high humidity areas rather than high vowel indices in arid regions. Nevertheless, it should be acknowledged that the intra-Australian trend contravenes those observed on the four major continents. It remains possible that the overall global pattern observed owes itself to coincidental trends on those continents. The Australian trend may hint at such a coincidental association, or it may simply hint at the problems of including analyses of continents with limited phylogenetic detail. The latter notion would seem to be more consistent with the cross-regional pattern in **Figure 5**, which suggests Australia is unexceptional at a less telescoped level.

It should be noted that, in some seminal typological work on the usage of correlational data, Australia has been grouped with New Guinea. In Dryer (1989), for instance, the five major global regions that are suggested for testing are Eurasia, Africa, North America, South America, and Australia/New Guinea. If that methodological tack is taken, we find that the association under examination does surface within each of the five regions. A beta regression between vowel index and humidity in Australia/New Guinea reveals a positive association (Pseudo *R*^2^ = 0.059, *p* < 0.00001). This association owes itself largely to the fact that many Australian language varieties are spoken in regions that are drier than New Guinea, and that the Australian vowel indices are generally low.

**FIGURE 6:**
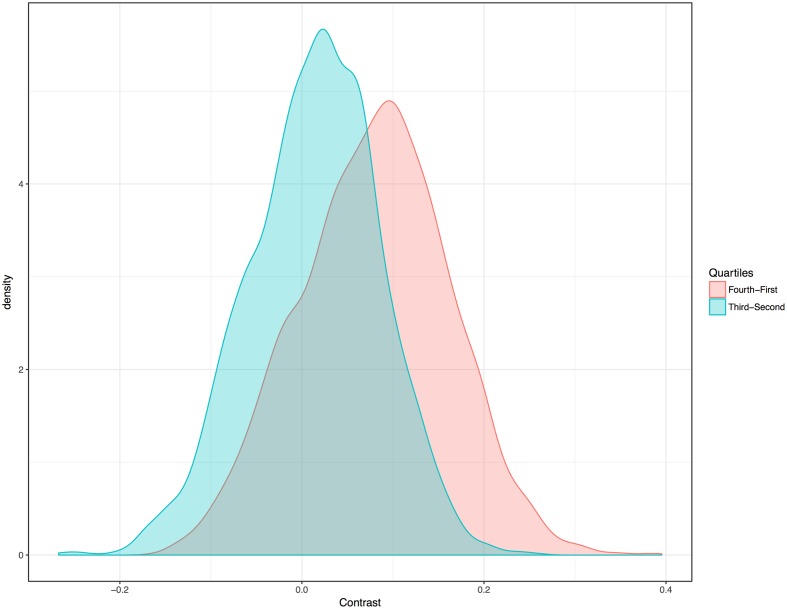
Density distribution (Gaussian kernel) for vowel-index contrasts of languages from different humidity quartiles. Each color represents 5000 contrasts. Each pairwise contrast was created from a new list, ordered by humidity and representing one randomly selected member of each family. The vowel index of one member of a given quartile was contrasted with the vowel index of a member of the other quartile.

Random sampling was also used to better elucidate the nature of the global association, in a manner more similar to that used in Everett et al. (2015) vis-à-vis tonality. One member of each language family was selected at random. The resultant list of 229 languages was then ranked according to humidity. For each of 5000 generated samples of this type, one member of the highest quartile of humidity was chosen at random (language a), and one member of the lowest quartile of humidity was chosen at random (language b). The vowel index of language b was then subtracted from the vowel index of language a. For each of 5000 additional samples, the same methods were applied except that languages were randomly selected from the 2nd and 3rd quartiles of humidity rankings, for each iteration. The net result of these 10,000 contrasts is depicted in **Figure 6**. (For verbose results, see “Materials and Methods.”) The 2nd and 3rd quartiles of phylogenetically controlled samples tend to have similar vowel indices, though the 3rd quartile generally has higher ones. In contrast, the lowest and highest quartile languages, in terms of humidity, differ more consistently with respect to vowel index. Languages with the lowest vowel indices are clearly likely to occur in dry regions.

Languages in dry regions simply do not exhibit very high vowel indices. The upper left quadrant of **Figure 3** is blank. Or consider that 55 of the languages in the driest quartile, for the entire dataset, have vowel indices two standard deviations or more below the mean vowel index. In contrast, zero languages in the driest quartile have vowel indices two standard deviations or more above the mean. This is consistent with the notion, requiring further exploration, that languages adapt to very dry air. Of course, even basic words are not immune to contact-based effects and languages moving into dry regions may come to have low vowel indices partly because their new neighbors do. Yet such influence would not explain why those neighbors consistently had low vowel indices in the first place.

One might object that the positive correlation between humidity and vowel indices does not necessarily imply lesser overall rates of vowel usage in drier contexts. Perhaps languages in drier climates use less vowels compared to consonants, but tend to have longer words so that their overall usage of vowels is not actually lower. This seems unlikely but, if it were the case, it would run counter to the suggestion that languages are adapting to ecological constraints. To test this possibility, the mean word length for each language variety was ascertained. Word lengths were based on the sum total of all consonants and vowels, for each of the 40 core words in the ASJP database. The average word length, by language variety, was 4.12 consonants and vowels (median 4.07), with some outlying language varieties differing in pronounced ways. (The lowest mean word length was 2.19, the highest was 9.42.) A very weak but positive correlation was observed between word length and humidity (Adjusted *R^2^* 0.033). So it is apparently not the case that languages in drier regions have lower vowel indices but compensate with greater word lengths. If that were so, there would be a negative correlation between word length and humidity.

Finally, it is worth separately examining large linguistic families to test whether the proposed association is evident within such groups. There are six aforementioned linguistic groups with more than 100 representatives (excepting “Australian,” just discussed), comprising over half the languages in the sample. Two of these have few if no representatives in the driest regions (Austronesian and Trans-New-Guinea). Within-family beta regressions suggest the tendency surfaces weakly within each of the four remaining major families. It is significant for Niger-Congo (*p* = 0.02), and Afro-Asiatic (*p* = 0.000), but not for Sino-Tibetan (*p* = 0.13) and Indo-European (*p* = 0.13). It is actually unclear whether any potential probabilistic effects of environment should accrue within families, but perhaps they do.

Such tests of large families hint at diachronic trends consistent with the influence of environment on language. But these tests of families are admittedly crude, treating families as flat structures. Much work is required to offer clear within-family diachronic support for the suggested influence. One potential approach is to implement the family bias method described in Bickel (2013), though such an approach would require binning individual languages according to vowel indices and humidity levels. Another possibility [suggested in Everett et al. (2016a)] would be to test linguistic migrations and within-family diachronic trends against climate models. This method would require fine-grained mappings of particular language families, utilizing Bayesian phylogenetic methods and/or trees established via more traditional comparative methods. In any case, the suggestion that climate impacts languages admittedly requires fuller exploration with more robust diachronic approaches. Unfortunately, however, such approaches will also face obstacles since only a handful of large linguistic families are mapped with sufficient confidence at present. Nevertheless, the vowel-usage data offered here may assist in such explorations. Given that the suggested influence is non-deterministic, and given that many factors are at play in sound changes, within-family trends across many linguistic taxa would ideally be considered.

I have suggested that the clearest potential explanation of the vowel-index/humidity association is that based on the deleterious effects of dry air on phonation. However, acoustically oriented accounts (e.g., Maddieson and Coupé, 2015) offer similar predictions. Those accounts are consistent with the fact that vowels are less common in drier regions. If the explanatory variable is in fact increased phonatory effort, as I am suggesting, perhaps we should see differences in the distribution of voiced consonants as well. Yet predictions for consonants are weaker since they generally require less phonation, and with less amplitude. Furthermore, the predictions of a phonation-oriented account are not uniform across manners of articulation. Consider nasals: While nasals are almost always voiced, they are made with a closed oral cavity and open nasal passageway. During inhalation, this configuration promotes humidification. So an account based on the effects of aridity on phonation makes no clear predictions for nasals. Nevertheless, the possibility of the effects of dry air on reduced voicing in consonants merits further exploration. As noted above, the ASJP database collapses some consonants according to voicing. This is particularly true of fricatives. Still, some limited explorations of voicing distinctions for consonants are possible. To that end, I examined the prevalence of voiced stops, nasals, rhotics, and laterals across the 4012 word lists. (The three latter kinds of sounds are generally voiced.) For each of these four consonant types, the total of the given consonant was divided by the number of all consonants and vowels, for each word list. Instead of a vowel index, then, this approach yielded a “voiced-stop index,” a “nasal index,” a “rhotics index,” and a “lateral index.” These indices were then tested against ambient humidity, as with the vowel index. Separate multivariate regressions, each with logit-transformed indices as dependent variables and humidity and language family as independent variables, were run. The results suggest that voiced stops are in fact slightly less common in more arid regions, even after controlling for language family in this manner (*p* < 0.0001). The same is true of rhotics (*p* < 0.001). No significant phylogenetically controlled patterns were observed for laterals and nasals. As just mentioned, though, the predictions of a desiccation-oriented account are unclear for nasals. Also, laterals may be voiceless and the voicing distinction is collapsed for laterals in the ASJP database. So the data do not lend themselves to clearly exploring degrees of phonation vis-à-vis consonants. And, as noted above, the predictions of a phonation-based account are much clearer for vowels. Nevertheless, this initial analysis of consonant data points intriguingly to the possibility of ambient pressures against the voicing of consonants as well, at least in the case of stops and rhotics. In the case of these consonants, though, it is unclear whether the patterns in question are independent of the more pervasive pattern associated with vowels, since vowels can influence the voice-onset-times of their consonant neighbors. Such issues require more substantive investigation with other databases.

## Discussion and Conclusion

The results presented here are consistent with the possibility that human sound systems evolve in accordance with environmental pressures. The association between low humidity values and low vowel indices is very strong, across languages (**Figure 2**) or language families (**Figure 3**). At the regional level, the evidence is generally supportive as well. The association surfaces on four of five major landmasess, though Australia is a counter-example. However, the Australian results are those that should be approached with the most caution for reasons observed above. While this is not the first study to suggest a potential environmental effect on languages, it is the first to do so without binning strategies that some scholars have found objectionable. It is also the first study to rely on sounds in actual words in 1000s of languages. The study has offered several controls for language families and areal influences, simultaneously. Yet the results are merely consistent with the idea that languages adapt, probabilistically and likely over long periods, to the influence of dry air on the larynx. Much work is required to understand this possible interaction. Some degree of circumspection is warranted since these data are correlational and the multifarious factors impacting language change are already known to be complex, and to interact in complex ways. And, of course, the possibility of a coincidental correlation remains and establishing causal links between correlated linguistic and extralinguistic variables is not straightforward. (Roberts and Winters, 2013) To paraphrase an old adage, though, while correlational data cannot establish causal relationships, they often gesticulate wildly in the direction of such relationships. Additionally, as researchers including myself have pointed out elsewhere, the traditional assumption that linguistic sounds are immune to ecological influence is problematic given the extent of ecological adaptability in human behavior (sometimes non-conscious), given that ecological adaptation characterizes the communication of other species, and given the dearth of research carefully examining this possibility. So while these results do not establish a causal influence of dry ambient air on language, they call for further consideration of this possibility and for continued exploration of this topic. They also demonstrate that global geo-phonetic correlations cannot be written off as the byproducts of convenient binning strategies.

As noted above, previous work has suggested that languages’ phonemic inventories have a greater ratio of consonants in colder (and therefore drier) climates. (Maddieson et al., 2011) A higher number of consonants in a phonemic inventory points to diversity of sounds in a language, which hints (but does not demonstrate) that the language may rely on consonants more in the speech stream. To examine a potential relationship between C:V ratios and vowel indices, the WALS data on C:V ratio complexity were cross-referenced with the new vowel index data. This yielded 262 languages, across a diversity of families and regions, with both vowel indices and C:V ratios. The C:V ratios in WALS are categorized on a scale from 1 to 5, with five representing languages with high ratios of consonants in their phonemic inventories. A weak but significant interaction of C:V ratio and vowel index was observed: Languages with high vowel indices tend to have lower C:V ratios, as we might expect. (pseudo *R^2^* = 0.029, *p* = 0.005). An effect of humidity on C:V ratio was also observed, with higher C:V ratios associating with drier regions (adjusted *R*^2^ = 0.04, *p* = 0.001). However, that interaction is quite modest when contrasted to the interaction of humidity and vowel index. (Given the limited number of languages with known C:V ratios and known vowel-indices, phylogenetic and areal controls could not be applied.) These findings suggest that vowel indices may more clearly reflect potential climatic effects on language, when contrasted to phonemic inventory data. It is hoped that vowel indices offer a useful new sort of data to be used in the further exploration of this topic.

In a similar vein, previous work with smaller, binned data sets has suggested that languages in warm regions rely more heavily on CV syllables for acoustic reasons (Munroe et al., 2009). While the prevalence of such syllables in warm regions may have acoustic motivations, that prevalence may also be due to the laryngeal factors discussed above. The claim that there are more CV syllables in warm places is, practically speaking, very similar to the claim that vowel indices are higher in humid places. The core of the account offered by Munroe and colleagues is this: People in colder regions tend to be closer to their interlocutors during speech events, and so need not rely as much on vowels that are so sonorant. This is an interesting claim, though I am unaware of any ethnolinguistic data supporting it. Given that there are findings in laryngology demonstrating an effect of dry air on the vocal cords, I believe that the most direct potential explanation for the distributional findings here and in previous work on “acoustic adaptation” in speech is one that is grounded on the interaction of vocal-tract physiology and ambient air. Yet, while I submit that this is the most plausible account at present, acoustically oriented accounts cannot be ruled out (much like coincidental distributions cannot be ruled out). It should also be noted, though, that while vowel indices correlate positively with temperature, their association with humidity is more pronounced. (See discussion of temperature data in “Materials and Methods.”) This suggests the former association may be epiphenomenal and would seem to further weaken the likelihood of an acoustically driven account.

The weak correlation between higher C:V ratios and lower vowel indices points to a crucial caveat required of the findings in this paper: Correlations that have previously been found via the usage of binning strategies should not be taken as independent support, alongside these, for the environmental adaptation of languages. For example, the apparent avoidance of complex tone in dry environs may be related to the association observed here since tone is conveyed via vowel pitch alterations. The ASJP database does not encode tonality, but future work could explore how interrelated these two findings are. It may be the case that the tonality findings are in a sense epiphenomenal, i.e., languages may come to rely less on tone when tone-carrying segments are less preponderant. Conversely, though, in some cases vowels may be more likely to be elided if they do not carry suprasegmental information. These sorts of tentative possibilities point to the difficulty of disentangling the interrelated patterns observed here and in previous work on tonality. Along the same lines, in Everett (2013) I suggested that ejective consonants might be more frequent at higher elevations because compression of the oral cavity is facilitated by reduced ambient air pressure. That is still an untested possibility, but it is also possible that the prevalence of ejectives in some regions is a byproduct of the relatively high frequency of consonants in arid ecologies. I am not making that claim here, instead I am simply pointing out that all these correlations described in the literature are potentially interrelated and cannot be taken as independent support for possible ecological effects on speech. Yet it is also true that the observed correlations run in the same direction, so to speak. So all these patterns are potentially explainable via one main effect of aridity on language. The results presented here, based on continuous variables, are likely the clearest signs of such an effect so far uncovered. But they are still just signs, guideposts at the beginning of an exploration. This exploration may eventually discover another variable that has not been considered in this work. Such a discovery could point to an indirect relationship between language and environment, rather than the direct one postulated here.

As I have pointed out previously (with Damián Blasi and Seán Roberts), one issue that requires further exploration is the way such patterns could surface over time. What kinds of mechanisms may actually motivate the distributional pattern described here? Are vowel elision and vowel epenthesis slightly more and less likely, respectively, to occur in very dry regions? Are innovators of sociolinguistic change more likely (however, slightly) to rely on easier-to-articulate lexical variants with reduced vowels, in dry regions? Are elderly speakers more likely to produce easier-to-articulate variants, given that they seem particularly prone to the effects of humidity on phonation? (Sundarrajan et al., 2017). Given that vocal-tract maladies like laryngitis are more prevalent in dry environments, are some sociophonetic variants more likely to be selected for, probabilistically over centuries, in such regions? As has been asked before, are speakers of tonal languages (particularly second-language speakers) slightly less likely to precisely replicate multiple level and/or contour pitches because of increased jitter rates in very dry contexts? Such questions hint at possible avenues of research. While linguists have carefully documented many kinds of sound change, it remains to be seen how an ecological factor that impacts the vocal cords could act in concert with known processes of diachronic change in or across languages. Finally, it is also possible that acoustic/perceptual factors also play a role in a potential language-ecology interaction, though I have expressed skepticism toward that possibility here. In short, much work is required to better understand the possible language-environment interaction.

The environment has recently been shown to impact languages in a way once-dismissed by some: languages in cold regions are more likely to express a distinction between “snow” and “ice,” when contrasted to languages in warm regions. Despite previous anecdotally based claims for and against the generalization, it was uncovered after careful sampling of many languages (Regier et al., 2016). As the authors of that study note, it is only when considering a probabilistic, rather than deterministic, connection between language and the environment that such patterns emerge. This conclusion would appear to hold not just with respect to some human words, but also with respect to human sounds. These new results are suggestive of another kind of probabilistic interaction between the environment and language. But the results are just pointing for now, and more research is required to find out exactly where they are pointing.

## Materials and Methods

Data and code in Supplementary Material. Coding and analysis conducted with R.

All *p*-values are two-tailed.

The forty basic concepts described by the word lists are presented here: https://en.wikipedia.org/wiki/Automated\_Similarity\_Judgment\_Program.

Vowel indices were obtained for each word list via a function that summed the vowels in a given list and then divided this sum by the total number of consonants and vowels in the list. The script for this function was based on the transcription conventions used for the ASJP database, to ensure that all vowels and consonants were counted and that secondary characters were excluded (Brown et al., 2008). Tone and length are not encoded in the database. Nasal and oral vowels were treated the same for the purposes of this study. In the ASJP database, some syllable types are simplified for transcription. In particular, CV7C, CVhC, CVxC, and CVXC syllables are reduced to CVC. (The *7* is a glottal stop, the *h* is a glottal fricative, the *x* is a velar fricative, and the *X* is a uvular fricative.)

As an example of how the vowel index is calculated, consider the Pirahã words in the ASJP database. There are 100 words in this case. Here is the list, with the words separated by commas: ti, gi7ai, tiatiso, gisai, gaihi, kaoi, go, ogiagao, aibai, hoi, hoi, ogi, pi7i, oihi, ipoihi, igihi, iti7isi, pibigi, giopai, tihihi, aoisi, tai, ipi, soi, isigihi, bipai, ahiai, sitoi, isapai, igai, isitai, apaitai, apapai, kosi, itaoi, kaopai, aitoi, ipopai, opoi, aosi, ko7otai, boasai, bogai, iosi, ibioi, ita7ipi, ohoai, abi, obi, aobisai, ko7o7as7∼aga, aiti, koabai, oabai, pibai, kobababopi, iho, hoagi, aitahoi, abaipi, ipopao, hoai, gai, hisi, kahaixai, ogihiai, pi, pi, a7ai, tahoasi, bigi, hoa7ai, hoa7ai, hoai, hoati, hoagaipi, agi, bigi hio7o7iai, bisi, ahoasai, bisi, kobiai, kopaiai, ahoai, hoai, agi, kabi, asi, ba7ai, hioi, and kasi.

There are 300 instances of the three Pirahã vowels. There are 464 total vowels and consonants. So the vowel index for this language is 300/464 or 0.64655.

Two symbols in the ASJP database, ∼ and $, are used to denote monosegmentality. Since this analysis was concerned with phonetic strings, these symbols were ignored. Even consonants with very short duration, like instances of prenasalization, were considered relevant. In some cases of definitive coarticulation, this choice would lead to slightly higher consonant counts. Since the goal here was to count all phonetic units, this choice was well motivated. In any case, this choice seems to have little impact on the overall results. To be sure of this, a separate analysis was run in which ∼ and $ were factored into the vowel index. In this analysis, each instance of ∼ reduced the consonant count of a list by one. Each instance of $ reduced the consonant count of a list by two. The vowel indices obtained through this method did not differ appreciably in most cases, and the key pattern evident in **Figure 3** is very similar regardless of which approach is taken with these symbols. With this slightly different approach to the vowel index, a beta regression analyzing the median vowel index (by family) according to median humidity (by family) reveals a pseudo *R^2^* of 0.257, *p* < 0.0000001.

Specific humidity values were originally gathered by Seán Roberts for a previous study (Everett et al., 2015). The data were obtained by averaging six decades worth of specific humidity values from the National Oceanic and Atmospheric Administration. Specific humidity is the measure of water content in the air, as a ratio of all water and air. Like absolute humidity, it is a much better indicator of water content than relative humidity (Maddux et al., 2016). Mean annual temperature data were obtained via the Bioclim package in ArcGIS by Justin Stoler, a colleague at the University of Miami.

The 4012 word lists used for the bulk of the analysis represent 2632 unique ISO codes that could be cross-referenced with the humidity data. This suggests that many ISO codes are represented with word lists for more than one dialect, a point supported by visual examination of the data set. Using ISO codes for cross-referencing ensured that dialects of the same language were tied to the same geographic region (which they are in most cases anyhow). This way, the dialects’ climate data were more characteristic of their history rather than recent migrations.

The shapiro test was used to examine whether vowel indices, humidity, and temperature represented normal distributions. None did, at least in part because most cultures are found in equatorial regions. So the median values are presented in the summaries of these variables, and analyzed alongside means in all cases.

Results of beta regression of means for all families in **Figure 3**: Log-likelihood = 371.8 on 3 Df, pseudo *R^2^* = 0.286. Results of beta regression of medians for all families: Log-likelihood = 365.5 on 3 Df, pseudo *R^2^* = 0.267. Results of beta regressions of families’ median vowel index and median humidity values, by continent: Africa = pseudo *R^2^* = 0.311, *p* = 0.001. Eurasia = pseudo *R^2^* = 0.293, *p* = 0.0003. North America = pseudo *R^2^* = 0.093, *p* = 0.029. South America = pseudo *R^2^* = 0.208, *p* = 0.00002.

For the one-random-language-per-family samples, the mean slopes and *R^2^* values for 1000 linear regressions per region were 6.27/0.23, (world), 4.96/0.21 (Eurasia), 6.83/0.25 (Africa), 3.51/0.13 (North America), and 5.66/0.17 (South America).

For the top/bottom quartile contrasts of languages randomly selected from families, by ordered humidity: In 893/5000 simulations, the languages from the bottom quartile of humidity had higher vowel indices than those from the top. For 3/5000, they were the same. For 4104/5000 the vowel indices of the highest humidity quartile were greater. For second/third quartile contrasts: In 2041/5000 simulations, the languages from the second quartile of humidity had higher vowel indices than the third quartile. For 4/5000, they were the same. For 2955/5000, the vowel indices of the third humidity quartile were greater than those for languages from the second quartile.

For the beta regression of 6901 language varieties’ vowel index and temperature values: pseudo *R^2^* = 0.099, *p* = 0.0000. This regression is less explanatory than that in **Figure 2**, suggesting that humidity is a better predictor of vowel index than temperature. A multiple regression found that temperature is a significant predictor of logit-transformed vowel indices (*p* = 0.00005), even after including linguistic family as an independent variable.

For the investigation of consonants: A nasal index, a voiced-stop index, a rhotic index, and a lateral index were each calculated separately. Some zero values were obtained since some word lists lack one of the consonant types. These zero values were raised to the lowest non-zero values for each respective index, in order to allow for logit-transformation of the values.

## Author Contributions

The author confirms being the sole contributor of this work and approved it for publication.

## Conflict of Interest Statement

The author declares that the research was conducted in the absence of any commercial or financial relationships that could be construed as a potential conflict of interest.
