# Supplementary material for: Languages in Drier Climates Use Fewer Vowels
Source: Front Psychol. 2017 Jul 27;8:1285. doi: 10.3389/fpsyg.2017.01285 (PMC5529419; doi:10.3389/fpsyg.2017.01285)
Supplement: Supplementary file 1 [file Data_Sheet_1.ZIP › SI/SIFigure1.docx]

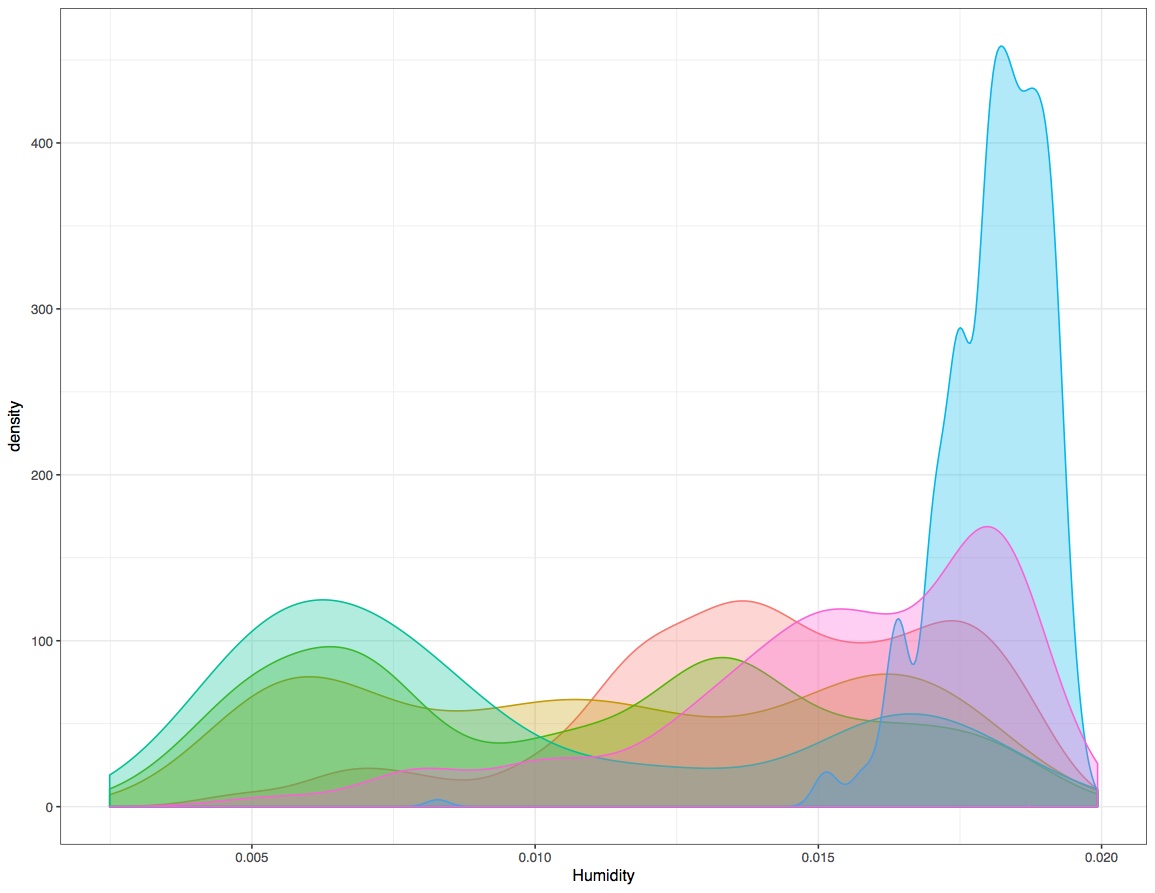


SI Figure 1. Density distribution of the six “regions”. The Pacific region, encoded in blue, has uniformly high humidity values.
